# Evidence-Based Learning Strategies in Medicine Using AI

**DOI:** 10.2196/54507

**Published:** 2024-05-24

**Authors:** Juan Pablo Arango-Ibanez, Jose Alejandro Posso-Nuñez, Juan Pablo Díaz-Solórzano, Gustavo Cruz-Suárez

**Affiliations:** 1 Centro de Investigaciones Clínicas, Fundación Valle del Lili, Cali, Colombia; 2 Departamento de Anestesiología, Fundación Valle del Lili, Cali, Colombia; 3 Unidad de Inteligencia Artificial, Fundación Valle del Lili, Cali, Colombia

**Keywords:** artificial intelligence, large language models, ChatGPT, active recall, memory cues, LLMs, evidence-based, learning strategy, medicine, AI, medical education, knowledge, relevance

## Abstract

Large language models (LLMs), like ChatGPT, are transforming the landscape of medical education. They offer a vast range of applications, such as tutoring (personalized learning), patient simulation, generation of examination questions, and streamlined access to information. The rapid advancement of medical knowledge and the need for personalized learning underscore the relevance and timeliness of exploring innovative strategies for integrating artificial intelligence (AI) into medical education. In this paper, we propose coupling evidence-based learning strategies, such as active recall and memory cues, with AI to optimize learning. These strategies include the generation of tests, mnemonics, and visual cues.

## Introduction

e-Learning has revolutionized the way medicine is taught and learned through the use of different internet-based technologies that enhance education [1]. Among these technologies, artificial intelligence (AI) tools, especially large language models (LLMs), have notably garnered significant attention in recent years, given their promising implications for medical education. LLMs are algorithmic models that are trained by extensive data sets, and they have the capability to comprehend text and generate natural-language text in response to a given prompt (input). This allows for interactive engagement with these technologies in a conversational format akin to a “chat” [2,3]. One of the most known LLMs is ChatGPT (owned by OpenAI), and its latest version, ChatGPT-4, was recently released to the public.

Recent studies have demonstrated the great achievements of LLMs in relation to medical knowledge and reasoning, such as ChatGPT-4 scoring 90% when answering USMLE (United States Medical Licensing Examination)–type questions [4], ChatGPT-4 passing a neurosurgery written board examination [5], and ChatGPT outperforming physicians in terms of providing empathic responses [6]. The educational potential of this technology is immense, encompassing a wide variety of applications. These include but are not limited to tutoring (personalized learning), patient simulation, generation of examination questions, and streamlined access to information [7-9]. The revolutionary potential of LLMs has resulted in researchers and medical students exploring the integration of AI into medical school curricula [10,11]. The rapid advancement of medical knowledge and the need for personalized learning underscore the relevance and timeliness of exploring innovative strategies for integrating AI into medical education [12].

Although numerous publications have examined the implications of LLMs for medicine and medical education, few have explored, in detail, specific strategies whereby LLMs can be used to optimize learning. In this paper, we propose strategies based on active recall, mnemonics, and the use of ChatGPT-4 [13] and DALL·E 3 (through ChatGPT-4) for enhancing learning outcomes regarding factual knowledge and thus help fill this gap of information. These strategies include the creation of pretests and posttest quizzes, the development of mnemonics, and the use of visual cues and mnemonics. Pretests and posttests serve as effective tools for active recall—a proven method for improving memory retention. Mnemonics simplify complex information into more digestible and memorable formats. Visual cues provide a graphical representation of information, aiding in better understanding and recall.

## Active Recall–Based Strategies

Medical school requires a significant amount of time spent on reading. Research indicates that medical students typically dedicate an average of 1.5 to 6 hours per day to reading [14,15]. Moreover, teacher-centered lectures, which predominantly focus on passive learning, persist as one of the most used strategies despite challenges in the medical education community with regard to encouraging integration with active learning methods that enhance the retention and application of knowledge [16-18]. This may be because medical school students might prefer classic didactic lectures over demonstrations, small group discussions, feedback activities, group work (generating test questions and coming up with solutions to a problem), and other active learning methods that have been reported to better enhance memory and retention [19].

It is essential to adopt evidence-based strategies to enhance learning efficiency, especially considering the substantial academic workload and the ongoing reliance on passive learning methods. One such strategy is active recall, which involves actively retrieving information that was initially acquired passively through lectures, articles, or videos. This strategy is known to enhance learning significantly in comparison with passive learning strategies [20,21]. In this context, it is beneficial for readers to approach their reading proactively. This can be achieved by prefacing their reading with self-directed inquiries, understanding the main topics of the material, consistently formulating questions, and recognizing key concepts of high significance. Some related techniques include elaborative interrogation, which consists of answering “why” questions about a given concept to enhance medium- to long-term associative memory; self-explanation to relate new information to known information or explain steps taken for solving a problem to improve memory, comprehension, and transfer; and practice testing, which can improve memory, is not as time consuming, and can be applied at different times of the learning process [20].

**Figure 1.**
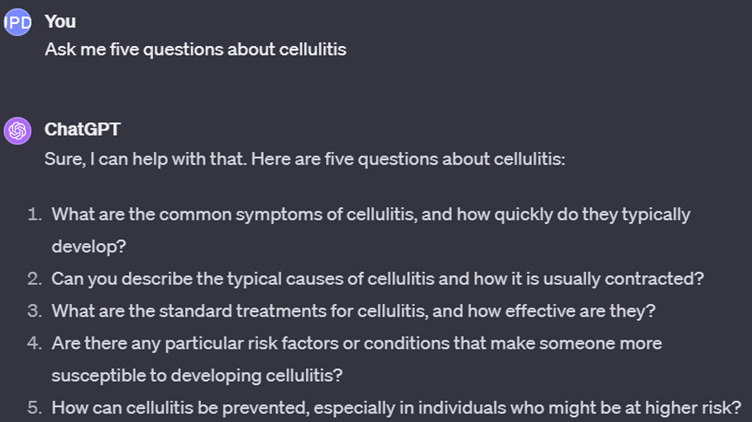
Example of the use of ChatGPT for pretesting.

One application of an active recall–based strategy involving the use of AI is illustrated in Figure 1, which shows ChatGPT being instructed to generate questions about cellulitis, as an example. Students are encouraged to attempt such questions before starting lectures or reading text. Answering questions before attending a lecture or reading a text (pretesting) is a strategy that enhances the learning process [22]. Interestingly, making mistakes during the study process can enhance learning by improving later memory; generating correct feedback; facilitating active learning; and stimulating the learner to redirect attention appropriately, especially when a mistake is followed by corrective feedback [23,24].

**Figure 2.**
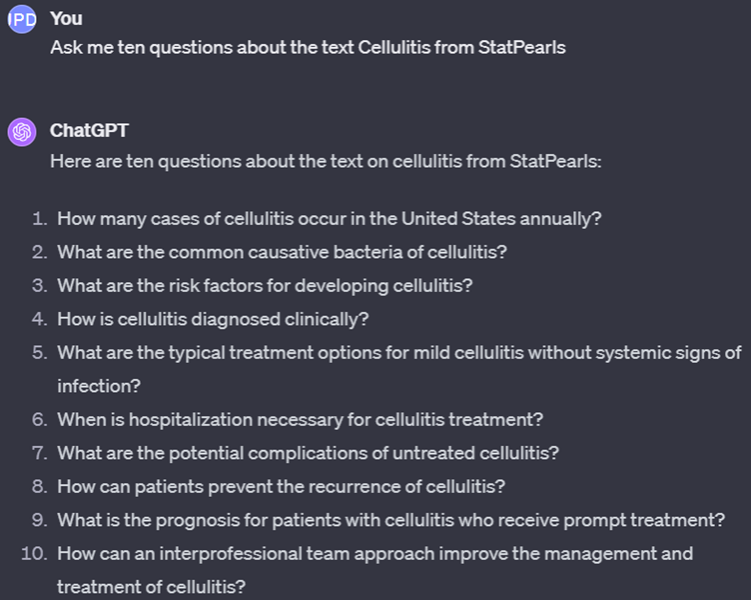
Example of the use of ChatGPT for creating a posttest.

Taking tests has been proven to enhance learning in various studies [25-28]. Repeated test-taking increases the transfer of learning [25] and improves long-term recall [28], and it even outperformed concept mapping for long-term retention in a previous study [26]. This strategy can be integrated with AI, as shown in Figure 2, which depicts our attempt to extract information from a *StatPearls* article on cellulitis [29] and request ChatGPT to generate relevant questions. The AI system can produce various question formats, such as multiple-choice, true-false, and fill-in-the-blank questions, when given the appropriate prompts. These questions may be stored and reviewed days or weeks after the initial review to successfully apply spaced repetition, which has been demonstrated to improve learning and the consolidation of knowledge [21].

**Figure 3.**
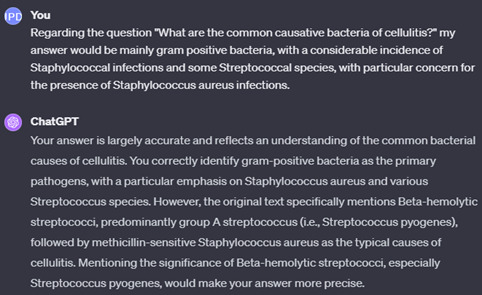
Feedback from ChatGPT on a partially correct answer to a question provided by ChatGPT.

**Figure 4.**
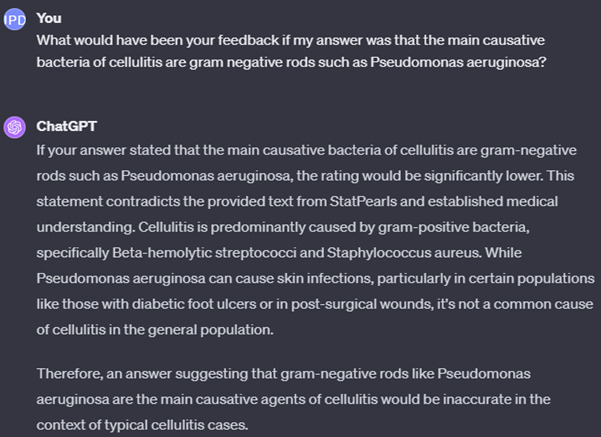
Feedback from ChatGPT on a wrong answer to a question provided by ChatGPT.

By using this method, one can input answers to questions and prompt ChatGPT to evaluate the answers’ accuracy against the provided text. For instance, using questions from Figure 2, we tested ChatGPT’s response by answering a query about common causative bacteria of cellulitis. We intentionally incorporated broad, correct concepts (gram-positive bacteria) and specific yet erroneous details (emphasizing staphylococci, particularly *Staphylococcus aureus*, as primary causatives instead of the correct streptococci) (Figure 3). ChatGPT feedback was tested again to contrast it with the feedback on a completely wrong answer (Figure 4).

## Memory Cues

Memory cues are learning strategies in which a process of metacognition transforms information in a way that makes the information easier to recall or understand. Cues can be self-generated or generated by external agents, other people, or AI. Evidence has long suggested that self-generated cues are superior to cues generated by other people [30,31]. Nonetheless, there is available evidence that indicates that memory cues generated by others can still enhance recall [32].

Memory cues are effective because they make difficult-to-remember information into something simpler or meaningful, which facilitates recall [32,33]. For example, a classic memory cue in medical school for remembering descriptors of pain is the use of the mnemonic “SOCRATES” (site, onset, character, radiation, associations, time course, exacerbating factors, and severity). In this context, the name of the great philosopher is repurposed to recall how to properly assess pain in a patient, with the name becoming an acronym. In a recent meta-analysis, a statistically significant effect was found for cueing decreasing the learners’ perceived cognitive load and promoting learning outcomes, namely retention, and the transfer of knowledge [34].

Other modalities of memory cues that are commonly used include pictures, short stories, songs, and rhymes [32]. Evidence indicates conflicting conclusions regarding the superiority of a specific modality of cues over another. For instance, in a study conducted by Pearson and Wilbiks [35], the authors attempted to evaluate the effect of the number of self-generated memory cues and aimed to test the findings of previous research that showed that the use of multisensory memory cues (ie, audiovisual cues) had a greater effect on recall than the use of one modality (ie, either visual cues [written words] or auditory cues [spoken words]). Their findings were that a greater number of cues led to higher recall, with statistical significance, but the modality of the cues did not have an effect on recall.

As previously indicated, one way to enhance the creation of an effective mnemonic is by using a common word as a cue to recall information [33]. This is one of the various ways that learners attempt to encode new vocabulary, abstract concepts, and master knowledge.

**Figure 5.**
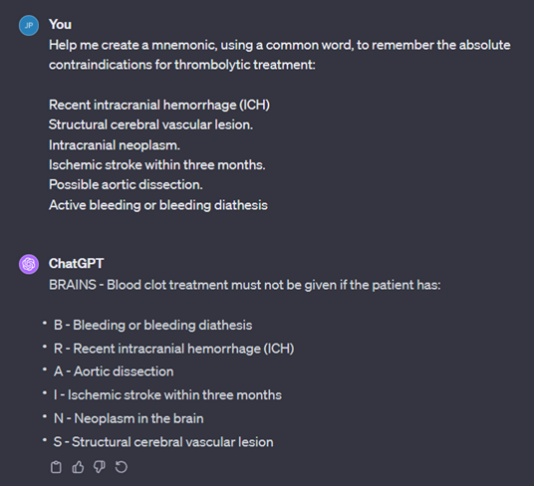
Acronym created by ChatGPT.

**Figure 6.**
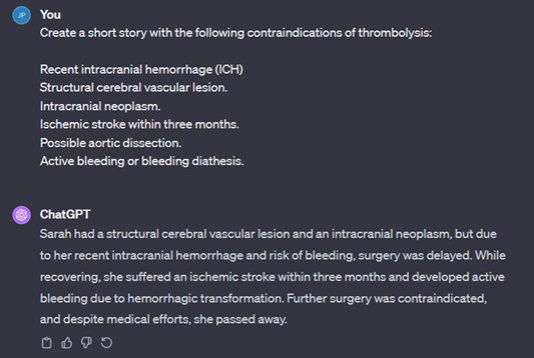
Short story created by ChatGPT.

**Figure 7.**
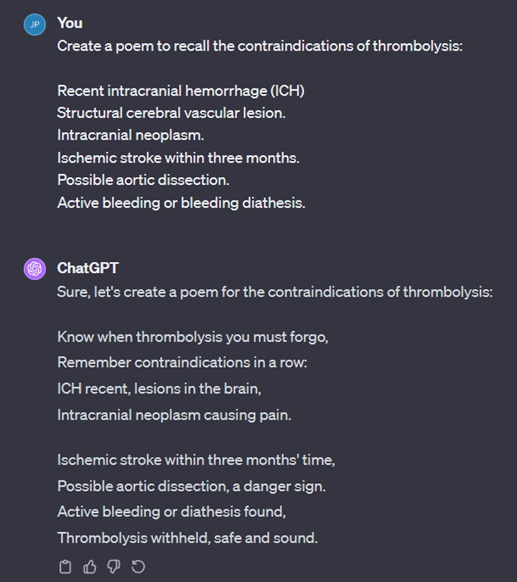
Poem created by ChatGPT.

Figure 5 is an example of ChatGPT generating a mnemonic, using the word “brains” to recall the absolute contraindications of thrombolysis. Other examples are shown in Figure 6, in which ChatGPT creates a short story, and in Figure 7, in which ChatGPT creates a poem.

## Visual Mnemonics

A visual mnemonic or cue is a tool that uses visual imagery to improve the recall of information. This differs from verbal mnemonics, which use words, phrases, or songs, as visual mnemonics use pictorial cues to forge memorable links. Their effectiveness stems from the incorporation of visual representations, analogies, or symbolism, which fortifies the associations and makes them more distinct. Visual mnemonics aid in recalling abstract or intricate information and facilitate both the sequential and the immediate retrieval of memorized material [36,37]. The use of mnemonics can be highly useful for learning difficult or abstract information [30], which is often found in the field of medicine [38]. Multiple studies have demonstrated that using visual or pictorial mnemonics can enhance learning outcomes [35,39].

**Figure 8.**
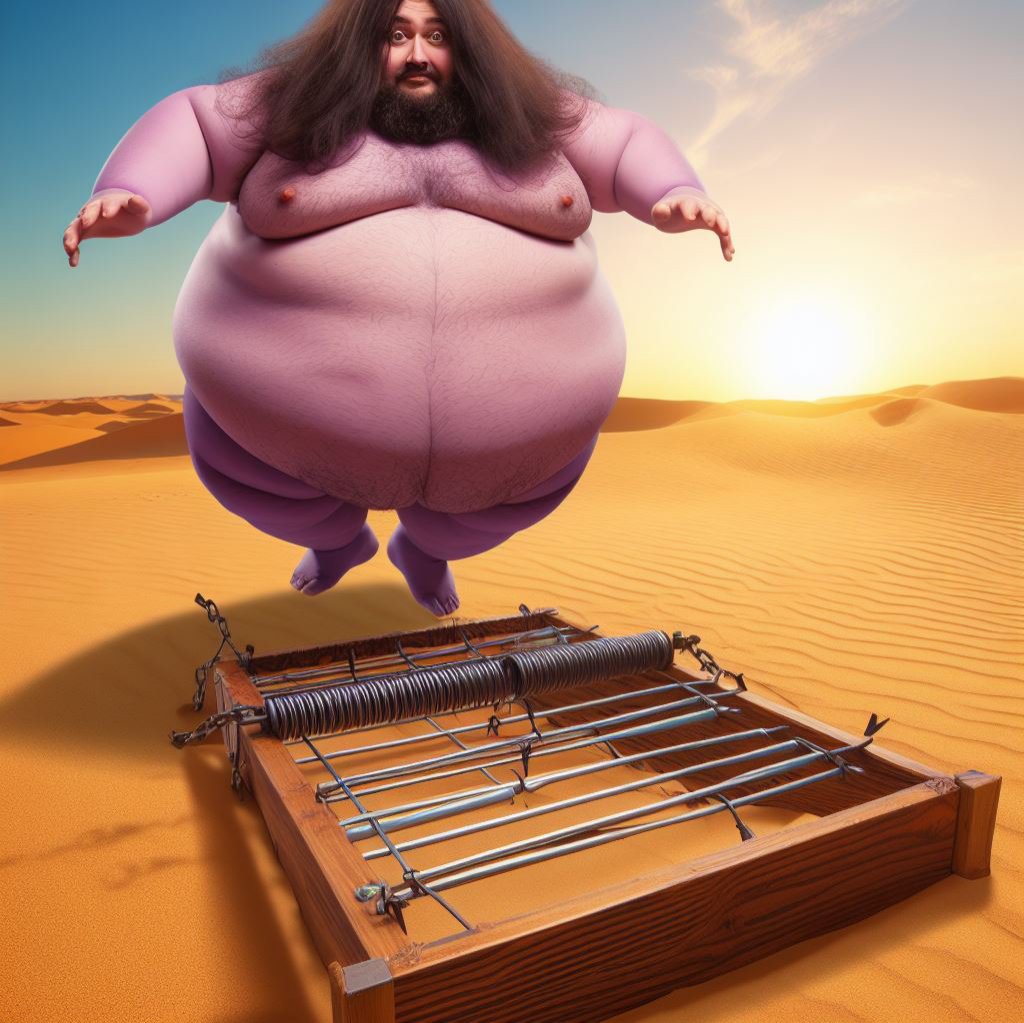
DALL·E 3 creation with the prompt “Fat purple man with long hair falling into a trap in a dry desert.”

DALL·E 3 is an AI system created by OpenAI that generates images based on prompts provided by the user and can be used for the creation of visual mnemonics. An example is given in Figure 8; a prompt was given to DALL·E 3 to create an image. For this example, which we created via DALL·E 3, the prompt “Fat purple man with long hair falling into a trap in a dry desert” was used to help recall some important features of hairy cell leukemia. “Fat” was used to recall the massive splenomegaly seen in patients with this condition; “purple” was used to make an association with lymphocytes, which are commonly seen as purple cells via hematoxylin and eosin staining and are involved in the pathogenesis of this neoplasm; “long hair” helps with recalling the filamentous projections of cells in hairy cell leukemia; “trap” was used to remember that this disease stains positively in tartrate-resistant acid phosphatase staining; and “dry desert” was used to recall that bone marrow fibrosis leads to dry tap on aspiration.

## Discussion

### Active Recall

Active recall is a highly effective learning strategy and significantly outperforms passive restudying when it comes to certain learning outcomes, such as conceptualization and long-term retention [21]. It has yielded better evaluation testing performance than traditional studying or rereading. Kornell et al [22] reviewed recall when participants were presented with fictional and nonfictional information, modifying the time for pretesting and read-only strategies. The testing strategy yielded a greater amount of correct answers than the read-only strategy, with statistical significance when equal or more time was allocated to the testing condition when the final test was performed more than 24 hours after the learning exercise, as well as in the fictional topic scenarios (*P*<.01). Other studies supporting the use of pretesting have been reported [22,40,41]. This highlights the role of pretesting in learning new information.

The benefits of active learning through testing have also been supported by other authors. Butler [25] tested students’ recall ability when they were either passively restudying or studying via repeated testing. Butler [25] found that repeated testing resulted in better performance on a recall test than passive learning strategies and concluded that repeated test-taking increases the transfer of learning. In another study performed by Karpicke and Blunt [26], when retrieval practice (testing) was evaluated against passive learning strategies and even concept mapping, it proved to be better for verbatim and inference question answering, resulting in an improvement of about 50% in long-term retention scores (*d*=1.50; *F*_1,38_=21.63; η_p_^2^=0.36). Additionally, the superiority of retesting over passive restudying for long-term retention has even been proven in a randomized controlled trial, wherein pediatric and emergency medicine residents were randomized to study the same text passages either via testing or repeated studying (ie, rereading). They were then tested on day 1, week 2, week 4, and month 6. The test results showed that the scores of participants who studied via testing were, on average, 13% higher than the scores of participants who performed repeated studying (*P*<.001), with an effect size of 0.91 [28]. Spaced testing (taking tests on different days between study sessions) has an even better effect on retention, long-term memory, and evaluation performance than repeated test-taking [27]. On the other hand, research indicates that spaced repetition (regardless of whether studying is done actively or passively) promotes more efficient and effective learning [42].

The previously mentioned studies highlight the importance of leveraging evidence-based techniques for studying rather than passive learning strategies. As we exemplified, these strategies can be coupled with AI. This approach addresses the limitation of relying solely on teacher-provided tests or textbook tests [20]. Moreover, ChatGPT is available on different platforms (web application and mobile app), and it can save chats (interactions) across these platforms. Therefore, students can easily access ChatGPT wherever it is needed and space their study sessions. Further, self-testing with AI reduces the pressure of graded assessments and leverages errors as learning opportunities [23,24], which research has shown to be particularly effective when the learner is confident in their incorrect answers [23]. This could be related to the effect of feedback.

Several articles on active recall and learning emphasize the role of feedback in enhancing learning processes, which is a characteristic that passive studying lacks. Roediger and Butler [27] compared traditional studying with test-taking studying without feedback and test-taking studying with differently timed feedback to determine whether test-taking studying results in better retention and whether retention is enhanced by feedback. They tested participants at different times and highlighted the efficiency of test-taking as a studying strategy, which was superior to that of traditional reading and restudying (22%, 32%, and up to a 43% difference between traditional studying and test-taking studying without feedback, test-taking studying with immediate feedback, and test-taking studying with delayed feedback, respectively). Since feedback has a clear impact on learning, especially when coupled with active recall strategies, non–AI-mediated active learning strategies could be limited by the lack of opportunities to offer feedback. Feedback generally comes from a reliable source, such as a teacher or an expert, or is obtained through an appropriate literature search, which can be time consuming. Sometimes, it is not possible to have the timely intervention of a teacher or an expert if there is a lot to study, and it may not be possible for a student to conduct a proper literature search if the student is new to a given topic. Furthermore, there are different types of feedback; some feedback is self-directed (ie, obtained through an introspective process). Feedback in the learning process can be used to enhance or develop skills for setting goals, monitoring one’s own learning process, and assimilating input (feedback) toward enhancing performance [43]. All of these are important skills to have when one attempts to obtain feedback on their own, such as when using LLMs for feedback.

AI can enhance medical education by offering feedback and explanations to clarify incorrect responses, thereby increasing study efficiency. By using AI tools like ChatGPT, students can receive detailed feedback on their answers, including the identification of errors and the provision of correct information, as we have shown. ChatGPT presents promising implications in providing technically accurate medical feedback, given the exceptional knowledge it has exhibited, as we previously described. This process, however, should not replace thorough literature research or foundational knowledge acquisition. AI models can also serve as tutors to facilitate discussions on specific knowledge areas, similar to existing models in other fields, such as Khan Academy’s Khanmigo, which serves as a fully personalized tutor [44]. One limitation of AI systems like ChatGPT is their character limit for inputs, which can be managed by breaking text into sections or using multiple prompts. Additionally, web searching is only available with ChatGPT’s paid subscription; for the free version, one should provide ChatGPT with the reference text by copying and pasting it. Further research is needed to explore the potential of AI-assisted tutors in medical education, especially in education on basic subjects.

### Memory Cues

Memory cues can be used as effective learning tools that ease the studying experience for a student attempting not only memorization but also mastery of complex concepts and new vocabulary. Evidence has described the superiority of self-generated memory cues over cues created by others [30,31]. The explanations behind the superiority of self-generated memory cues are (1) the generation effect of creating such cues, that is, the act of generation requires significant cognitive effort, which boosts memory, and (2) the cue selection process itself and the consequential metamnemonic effect. This means that students who identify the learning formats that work best for them are able to create their own memory cues by using a modality that is tailored to their needs [31]. For example, if a student prefers to have a visual representation of the ideas that they are attempting to memorize, they might lean toward the creation of mnemonics that create a mental picture to integrate information. Tullis and Fraundorf [31] proposed evidence that the benefits of self-generated cues come in great part from the correct selection of a cue from a list of candidates. If students can create multiple cues, they can, with greater effectiveness, select the cue that best benefits retrieval. Tullis and Fraundorf [31] further suggested that allowing a learner to select from multiple options of cues requires less cognitive work, takes less time, and may not hinder memorization.

As we described, the act of generation is effortful and may be time consuming. AI tools like ChatGPT can help students as a result of their seemingly tireless and effortless generative capacity. In addition, these tools can create multiple cues with different modalities (textual-based cues and pictorial cues) when prompted to do so, thereby allowing learners to focus on understanding the material and selecting the most appropriate cue that fits their educational needs. The downside to the use of this method is that multiple attempts may be required for ChatGPT to produce a mnemonic that is subjectively good or fitting enough for a particular student. In addition, it is our opinion that these tools are best used when the user has an idea of what they should learn or memorize, and the user should prompt the AI tool to create a mnemonic device that facilitates the recall of the information they wish to encode. This is because there is abundant evidence of ChatGPT not only making errors but also blatantly providing false information [45], which is known as “artificial hallucination.”

### Visual Mnemonics

Previous research has explored the effectiveness of visual mnemonics in improving learning outcomes. An experimental study that compared pictorial mnemonic use to traditional study methods found that pictorial mnemonics aid in learning from text passages by improving the recall of factual knowledge and long-term memory retention in college students [46]. Additionally, a randomized trial compared audiovisual mnemonics against traditional text-based learning for retaining medical knowledge; participants who used mnemonics demonstrated significant improvements in free-recall tests, with scores improving by 65%, 161%, and 208% immediately, after 1 week, and after 1 month, respectively, when compared to those who used text materials (*P*<.001). Moreover, the group that used mnemonics outperformed the group that used text materials by 55% in a 1-week–delayed multiple-choice test that focused on higher-order thinking (*P*<.001) [47]. In a comparative study of visual mnemonics versus traditional lectures for learning the porphyrin pathway, there was no significant difference in quiz scores immediately or 1 week after the intervention; however, the mnemonic group exhibited a 20% higher score 3 weeks later (*P*=.02) [48]. In another randomized trial that compared story-based audiovisual mnemonics with traditional text reading for memory retention among medical students, the audiovisual mnemonics group demonstrated significantly better performance in multiple-choice tests immediately after the intervention (*P*=.04), as well as at 1 week, 2 weeks, and 4 weeks after the intervention [49]. These results underscore story-based mnemonics’ superior effectiveness in enhancing immediate and long-term memory retention in medical education. Although there is some variation in the visual mnemonic techniques across studies (eg, the studies by Yang et al [47] and Abdalla et al [49] used some audiovisual mnemonics), they consistently demonstrated that factual knowledge can be represented visually and that the use of this type of mnemonic enhances both the recall and long-term retention of knowledge, with large effect sizes.

The visual mnemonic proposed in our study highly resembles the strategy used in the experiment by Rummel et al [46], in which visual mnemonics were created from texts about psychologists, incorporating elements for recalling both the psychologists’ names and the key aspects of their theories. In our mnemonic, “long hair” aids in recalling the name of the disease (hairy cell leukemia), and the other elements in the image are used to help recall the disease’s main features. The Picmonic System, which uses mnemonics from a web-based educational platform [50] that was used in the studies by Yang et al [47] and Abdalla et al [49], also adopts the visual mnemonic approach by combining visual elements and storytelling to enhance the recall of information; this is also highly similar to our approach. Thus, using DALL·E 3 for mnemonic generation shows promise for improving different learning outcomes, such as test performance, long-term retention, and free recall. Future studies should experimentally investigate the effectiveness of visual mnemonics generated by text-to-image models in learning processes. A significant limitation of using DALL·E 3 for medical mnemonic generation is its restriction on explicit content, prohibiting prompts with terms like “blood.” By recognizing this limitation, knowledge area–specific text-to-image models can be developed to more accurately describe the information needed and enable the use of words that are commonly used in a knowledge area but are censored in current models. Another limitation is that creating stories that accurately reflect the intended factual knowledge for mnemonic cues can be complex, particularly for certain subjects. Effective prompt engineering techniques could help in creating more relevant and coherent visual mnemonics.

## Conclusions

LLMs, as a form of AI, are transforming the landscape of medical education. They offer a vast range of applications, and their potential has sparked discussions about integrating them into medical school curricula. Active recall–based learning strategies can be integrated with AI and can promisingly improve the recall and retention of information. This integration can be effectively applied by using AI to generate pretests and posttest quizzes. Memory cues, including self-generated mnemonics and mnemonics created by AI, can effectively simplify and transform complex information, thereby enhancing recall and optimizing learning. ChatGPT can create multiple types of memory cues, such as acronyms, short stories, and even poems. Moreover, AI tools, like DALL·E 3, can create images based on text and thus can be used to create visual mnemonics. However, crafting the right prompts can be challenging and time consuming, and results may vary. Thus, we believe that the use of new AI-based technologies, such as ChatGPT and DALL·E 3, is a highly useful strategy for learning, especially when these technologies are used with evidence-based principles. Further research is warranted to elucidate the impact of these strategies within the context of medical education.

## Abbreviations

AI: artificial intelligence
LLM: large language model
SOCRATES: site, onset, character, radiation, associations, time course, exacerbating factors, and severity
USMLE: United States Medical Licensing Examination
